# Isolation and identification of antagonistic *Bacillus amyloliquefaciens* HSE-12 and its effects on peanut growth and rhizosphere microbial community

**DOI:** 10.3389/fmicb.2023.1274346

**Published:** 2023-10-12

**Authors:** Huying Li, Chaohui Li, Xin Song, Jintai Li, Pengcheng Zhang, Fengxia Sun, Zhigang Geng, Xunli Liu

**Affiliations:** ^1^College of Forestry, Shandong Agricultural University, Taian, China; ^2^School of Nursing, Zibo Vocational Institute, Zibo, China; ^3^Key Laboratory of National Forestry and Grassland Administration on Silviculture of the Lower Yellow River, Shandong Agricultural University, Taian, China; ^4^Ministry of Agriculture Key Laboratory of Seaweed Fertilizers, Qingdao, China

**Keywords:** *Bacillus amyloliquefaciens*, plant growth-promoting rhizobacteria, microbial community, biocontrol, volatile compounds

## Abstract

The HSE-12 strain isolated from peanut rhizosphere soil was identified as *Bacillus amyloliquefaciens* by observation of phenotypic characteristics, physiological and biochemical tests, 16S rDNA and gyrB gene sequencing. *In vitro* experiments showed that the strain possessed biocontrol activity against a variety of pathogens including *Sclerotium rolfsii*. The strain has the ability to produce hydrolytic enzymes, as well as volatile organic compounds with antagonistic and probiotic effects such as ethyleneglycol and 2,3-butanediol. In addition, HSE-12 showed potassium solubilizing (10.54 ± 0.19 mg/L), phosphorus solubilization (168.34 ± 8.06 mg/L) and nitrogen fixation (17.35 ± 2.34 mg/g) abilities, and was able to secrete siderophores [(Ar-A)/Ar × 100%: 56%] which promoted plant growth. After inoculating peanut with HSE-12, the available phosphorus content in rhizosphere soil increased by 27%, urease activity increased by 43%, catalase activity increased by 70% and sucrase activity increased by 50% (*p* < 0.05). The dry weight, fresh weight and the height of the first pair of lateral branches of peanuts increased by 24.7, 41.9, and 36.4%, respectively, compared with uninoculated peanuts. In addition, compared with the blank control, it increased the diversity and richness of peanut rhizosphere bacteria and changed the community structure of bacteria and fungi. The relative abundance of beneficial microorganisms such as *Sphingomonas*, *Arthrobacter*, *RB41*, and *Micromonospora* in rhizosphere soil was increased, while the relative abundance of pathogenic microorganisms such as *Aspergillus*, *Neocosmospora*, and *Rhizoctonia* was decreased.

## 1. Introduction

Peanut (*Arachis hypogaea* L.) is an important cash and oil crop in China, as well as a rich source of protein, vitamins, minerals and medicinal active ingredients such as flavonoids and phenolic compounds (Li et al., 2021; Mallano et al., 2021). Due to the high economic benefit and limited planting area, peanuts are often intensively planted. Applying chemical fertilizer is one of the most commonly used ways to develop intensive agriculture (Ye et al., 2020). However, long-term excessive use of chemical fertilizers has caused many serious environmental problems, such as air pollution, soil acidification and food safety decline (Ning et al., 2017). Organic fertilizer has attracted more and more attention with the rapid development of green agriculture. However, the efficiency of organic fertilizer release is difficult to predict and control (Ding et al., 2019). Microbial fertilizer can not only increase the yield, improve fertilizer utilization rate and improve the quality of agricultural products, but also promote the formation of new microbial flora in crop roots and improve the soil environment. This has played a positive role in the development of green agriculture (Ziane et al., 2017; Liu et al., 2022). On the other hand, peanuts are susceptible to many diseases caused by fungi or bacteria, which leads to the decline of yield and quality (Liu et al., 2017; Sobolev et al., 2018). In order to prevent these diseases, different control strategies have been applied in the past decades, such as using fungicides, biological control and breeding resistant varieties (Liu et al., 2017). Until now, microbial control has been considered as one of the most promising and environmentally friendly methods to control plant diseases (Chen et al., 2019).

Plant growth-promoting rhizobacteria (PGPR) inhabits the rhizosphere or root of plants and can promote plant growth by various mechanisms, such as producing plant hormones, improving plant nutrient supply, inhibiting plant pathogens and changing soil physicochemical properties (Li et al., 2020; Wang et al., 2022a). On the other hand, many studies have shown that plant growth promoting rhizobacteria can also affect plant development and health by affecting the composition of soil microbial communities in plant rhizosphere (Ngalimat et al., 2021; Manh Tuong et al., 2022). Wang et al. found that *Bacillus velezensis* YYC could significantly promote the growth of tomato fibrous roots and significantly reduce the incidence of tomato bacterial wilt by increasing the activity of defense-related enzymes (Yan et al., 2022). *Bacillus amylolequefaciens* BChi1 and *Paraburkholderia fungorum* BRRh-4 significantly increased strawberry yield (up to 48%), and the contents of phenolic compounds, carotenoids, flavonoids and anthocyanins in strawberry fruit were significantly higher than those in untreated control (Rahman et al., 2018). Zhang et al. isolated *Tsukamurella tyrosinosolvens* P9 with phosphate solubilizing ability from tea rhizosphere soil significantly promoted the growth of peanut seedlings and increased the relative abundance of beneficial and functional microorganisms in peanut roots (Zhang et al., 2021).

In this study, *Bacillus amyloliquefaciens* HSE-12 was isolated from peanut rhizosphere, which can promote plant growth by producing siderophore, fixing nitrogen, dissolving phosphorus and potassium, and can also produce protease, cellulase and chitinase to inhibit fungal growth, and has the potential of biological control. The effects of HSE-12 on peanut growth, soil chemical properties, soil enzymes, rhizosphere microbial diversity and community structure were studied. It is expected to provide theoretical basis and strain support for the development of peanut special microbial fertilizer.

## 2. Materials and methods

### 2.1. Collection of soil samples and bacterial isolation

Plant growth promoting rhizobacteria were isolated from peanut rhizosphere soil in Yishui County, Linyi City, Shandong Province, China (35°40′05′ N, 118°42′45′ E). The soil suspension was prepared by adding 1 g of soil to 99 mL of sterile water and serially diluting 10 times. The isolated bacteria were inoculated with 200 μL of the dilution in Luria Bertani medium (LB) (tryptone 10 g/L; yeast extract 5 g/L; sodium chloride 10 g/L, agar 20 g/L) and then incubated in the plates at 28°C for 24 h. After purification the different colonies were selected by repeated purification in LB at least three times, depending on the shape, color, size, regular or irregular, convex or equal morphological characteristics of the colonies. The purified strains were stored in a 30% glycerol solution at −80°C.

### 2.2. Screening of antagonistic bacteria

The antagonistic abilities of the isolates were determined in dual-plate confrontation assays against the plant pathogens *Sclerotium rolfsii*, *Fusarium oxysporum*, *Rhizoctonia solani*, *Fusarium graminearum*, *Fusarium pseudograminearum*, *Alternaria alternata*, *Botryosphaeria dothidea*, *Bipolaris sorokiniana*, and *Cryphonectria parasitica*. After 4 days, the diameters of the zone of fungal growth inhibition around the bacteria were measured and recorded.

### 2.3. Identification of PGPR strains

The isolates were characterized by observing the phenotypic characteristics such as colony morphology, color, size, edge and Gram staining. In addition, the experiments of glucose oxidative fermentation, contact enzyme, nitrate reduction, starch hydrolysis, methyl red, V-P, gelatin liquefaction, citrate utilization, hydrogen sulfide, oxidase, and indole were carried out. All tests were made in triplicate.

16S rDNA and gyrB sequence analysis was used to identify the strain. The strain was inoculated in LB liquid medium and shaken at 37°C (200 rpm) for 24 h. Bacterial genomic DNA was extracted from each strain using the bacterial genomic DNA isolation kit DP302 (Beijing Tiangen Biochemical Technology Co., Ltd., China). Genomic DNA was extracted from each strain using 16S rDNA gene primers 27F (5’-AGAGTTTGATCCTGGCTCAG-3′) and 1492R (5’-TACGACTTAACCCCAATCGC-3′) for amplification. The reaction was performed with 25 μL of 2 × taq PCR mix, 1 μL of forward primer, 1 μL of reverse primer, 1 μL of template DNA and 22 μL of ddH_2_O for 50 μL. Reaction conditions: 94°C for 1 min, 33 cycles of 94°C for 20 s, 54°C for 20 s and 72°C for 2 min, followed closely by a final extension at 72°C for 10 min.

The gyrB sequence of bacteria was amplified by using primers G3F (5’-GAAGTCATCATGACCGTTCTGCAYGCNGGNGGNAARTTYGA-3′) and G3R (5’-AGCAGGGTACGGATGTGCGAGCCRTCNACRTCNGCRTCNGTCAT-3′). Reaction conditions: 94°C for 1 min, 35 cycles of 94°C for 20 s, 58°C for 20 s and 72°C for 2 min, followed closely by a final extension at 72°C for 10 min. Reaction products were qualified by 0.8% agarose gel electrophoresis and then sent for sequencing to Sangon Biotech (Shanghai) Co. The NCBI Blast server^1^ was used for comparison with sequences registered in the GenBank database. Phylogenetic trees were constructed using the Neighbor-Joining method in the MEGA (version 7.0) package and 1000 bootstrap replicates were used to evaluate the topology of the phylogenetic trees.

### 2.4. Determination of fungistatic characteristics of HSE-12 strain

Bacterial ability to produce extracellular antifungal metabolites and volatile organic compounds (VOCs) and antagonism to the plant pathogen *Sclerotium rolfsii* was tested using a double plate antagonism assay (Delgado et al., 2021). The type and relative content of VOCs produced by the antagonistic bacteria were determined and analysed by gas chromatography–mass spectrometry (GC–MS). The VOCs were collected using SPME with a polydimethylsiloxane/divinylbenzene (65 μm) fiber (Supelco Inc., Bellefonte, PA, United States) at 40°C for 30 min. The VOCs were analyzed using the Shimadzu GCMS-QP2010 gas chromatograph (70 eV, Shimadzu Co., Kyoto, Japan) equipped with a capillary column of Rtx-5MS (60 mm × 0.2 mm, film thickness 0.25 μm). The carrier gas was helium with a flow rate of 1 mL/min. The oven temperature of GC analysis was programmed as 40°C for 2 min at the beginning, raised to 160°C at 8°C/min and held for 2 min, then to 240°C at 15°C/min and held for 3 min. The mass spectrometer was performed in the positive electron ionization mode at 70 eV at 200°C and scan range of 45–500 m/z. The mass spectra of VOCs were compared with available data in the NIST17 Library to identify these compounds. In addition, extracellular hydrolases involved in the growth inhibition mechanism of the fungus, including chitinase, protease, cellulase and xylanase, were measured quantitatively or detailed steps refer to Fang and Chen (2018).

### 2.5. Determination of plant growth-promoting properties of HSE-12 strain

#### 2.5.1. Determination of nitrogen fixation capacity of HSE-12

Fresh cultures of the strains were spot inoculated on ashby medium (mannitol 10 g, KH_2_PO_4_ 0.2 g, MgSO_4_ 0.2 g, NaCl 0.2 g, K_2_SO_4_ 0.3 g, CaCO_3_ 5 g, agar 15 g, distilled water 1,000 mL), and incubated at 30°C for 3 d to observe the growth of colonies and the formation of hyaline circles. The determination of nitrogen fixation efficiency was referred to the method of Panneerselvam et al. (2021).

#### 2.5.2. Determination of potassium dissolving ability of HSE-12

Fresh cultures of the strains were spot inoculated on silicate bacterial isolation medium (sucrose 10 g, yeast paste 0.5 g (NH_4_)_2_SO_4_ 1 g, Na_2_HPO_4_ 2 g, MgSO_4_∙7H_2_O 0.5 g, CaCO_3_ 1 g, potassium feldspar powder 1 g, agar 15 g, distilled water 1,000 mL), and incubated at 30°C for 3 d to observe the growth of colonies and the formation of hyaline circles.Potassium-solubilization efficiency was estimated by flame photometry (Yaghoubi et al., 2017).

#### 2.5.3. Determination of phosphorus dissolving ability of HSE-12

Fresh cultures of the strains were spot inoculated on inorganic phosphorus bacterial screening medium (NBRIP, glucose 10 g, MgCl_2_ 5 g, NaCl 0.3 g, MgSO_4_·7H_2_O 0.3 g, MnSO_4_ 0.03 g, K_2_SO_4_ 0.3 g, FeSO_4_ 0.03 g, (NH_4_)_2_SO_4_ 0.5 g, Ca_3_(PO_4_)_2_ 5 g, agar 15 g, distilled water 1,000 mL), and incubated at 30°C for 3 d to observe the growth of colonies and the formation of hyaline circles. The solubility of the strains for inorganic phosphorus was determined using the molybdenum blue colorimetric method (Song et al., 2021).

#### 2.5.4. Determination of the ability of HSE-12 to produce siderophore

Fresh cultures of the strains were spot inoculated on CAS detection medium (CAS 60.5 mg, HDTMA 72. 9 mg, FeCl_3_·6H_2_O 2.645 mg, NaH_2_PO_4_·2H_2_O 295.25 mg, Na_2_HPO_4_·12H_2_O 1213.5 mg, NH_4_Cl 125 mg, KH_2_PO_4_ 37.5 mg, NaCl 62.5 mg, agar 9 g, distilled water 1,000 mL), and incubated at 30°C for 3 d to observe the growth of colonies and the formation of hyaline circles. Siderophores were quantified using spectrophotometry (Wang et al., 2022b).

### 2.6. Reisolation of HSE-12 from rhizosphere

The colonization of HSE-12 in peanut rhizosphere was studied by double antibiotic labeling method.The mutant of HSE-12 was obtained by inducing with rifampicin and spectinomycin. The induction method of antibiotic-resistant mutant strains was as described by Chen et al. (1995) with slight changes, and the specific methods were as follows. HSE-12 was inoculated into 50 mL of LB liquid culture medium without rifampicin, and cultured for 24 h with shaking at 30°C and 200 r/min. Inoculate the above bacterial liquid into LB liquid culture medium containing 5 μg/mL Rifampicin at the inoculation amount of 2%, and shake culture at 30°C and 200 r/min for 24 h. Then the bacterial liquid was inoculated into LB liquid culture medium containing 10 g/mL rifampicin with an inoculation amount of 2%, and cultured for 24 h with shaking at 30°C and 200 r/min. By analogy, the concentration of rifampicin was gradually increased from 5 μg/mL, 10 μg/mL, 20 μg/mL, 40 μg/mL, 80 μg/mL and 150 μg/mL to 300 μg/mL, and the strains resistant to rifampicin were obtained. The strains resistant to rifampicin were inoculated into LB liquid medium containing 300 μg/mL rifampicin and 5 μg/mL spectinomycin, and the double antibiotic-labeled strains resistant to both rifampicin and spectinomycin were screened. During the screening process, the concentration of rifampicin in LB liquid medium was kept at 300 μg/mL, and the screening method of spectinomycin concentration was the same as that of strains resistant to rifampicin, and finally both rifampicin and spectinomycin were obtained. The screened double-antibiotic-labeled strains were cultured alternately in LB solid medium and LB liquid medium without rifampicin and spectinomycin for 3 ~ 5 generations, and then cultured in LB liquid medium containing 300 μg/mL rifampicin and spectinomycin, respectively, to confirm the drug resistance stability of the strains, and finally the double-antibiotic-labeled strains with stable inheritance and resistance to rifampicin and spectinomycin were obtained. The mutant was cultured overnight in LB broth at 30°C and 200 r/min to 1 × 10^8^ cfu/mL. The bacterial suspension was centrifuged at 1000 × g for 10 min. Each pot of peanut was poured with 50 mL of bacterial suspension. Soil was recovered from peanut rhizosphere every 10 days to verify whether the selected strains could colonize peanut rhizosphere, and bacterial colonies in peanut rhizosphere were counted by plate dilution method.

### 2.7. Pot experiment

#### 2.7.1. Design of pot experiment and inoculation of HSE-12

A pot experiment was used to study the effect of strain HSE-12 on the growth of peanut seedlings. CK and HSE-12 strain treatments had 6 pots each, with 3 peanut seeds in each pot. The experiment had a no-inoculation control and an inoculation treatment with strain HSE-12. The peanut seeds were surface sterilized as described above. The HSE-12 strain was incubated in LB broth at 30°C for 48 h in an oscillating incubator at 200 rpm and centrifuged at 4000 g for 10 min; the organisms were resuspended in sterile ddH_2_O and adjusted to 6–8 × 10^8^ CFU/mL. Six sterilized peanut seeds were sown in 10 L terracotta pots. For the HSE-12 treatment, the roots of 1-week-old seedlings were inoculated with 20 mL of diluted HSE-12 suspension; control plants were inoculated with equal amounts of sterile water for the root system. All pots were irrigated once before sowing to ensure proper seed germination and then watered regularly during crop growth in accordance with agronomic practices.

#### 2.7.2. Sample collection

Peanut seedlings were pulled out separately after 2 months and plant biomass indices were determined. Careful collection of rhizosphere soil was carried out with 2 random replicates of each treatment mixed into 1 sample. The soil samples were passed through 2 mm sieve, well homogenized and stored at −80°C for microbial community structure analysis.

#### 2.7.3. Agronomic character index measurement

The main stem height (SH), the height of the first pair of lateral branches (LBH), the fresh weight (FW), dry weight (DW) of the above-ground parts and the number of pods (NP) were measured for 18 seedlings from each treatment.

#### 2.7.4. Assays of the antioxidant system and other enzymes

The activities of superoxide dismutase (SOD), catalase (CAT), peroxidase (POD) and polyphenol oxidase (PPO) activities, according to the method of Zhang et al. (2020).

#### 2.7.5. Determination of soil chemical properties and enzyme activity

Available nitrogen (AN) determination using diffusion method, available phosphorus (AP) using sodium bicarbonate extraction method, available potassium (AK) using flame spectrophotometry determination, the determination of organic matter (OM) using potassium dichromate oxidation outside heating method (Bao, 2013). The activities of urease, catalase, invertase and phosphatase are referred to the method of Wang et al. (2020).

### 2.8. Soil DNA extraction

To study the effect of HSE-12 strain on soil microbial community structure, soil genomic DNA was extracted from control and HSE-12-treated soil using a soil DNA kit (Omega Bio-Tek, China). The V3-V4 region of the bacterial 16S rRNA gene was amplified with bacterial universal primers 338F and 806R, and the fungal-specific primers ITS1F and ITS2R were used to amplify the ITS1 region of the fungus. High-throughput sequencing was performed by Majorbio Bio-Pharm Technology Co., Ltd. (Shanghai, China) on the Illumina Miseq PE300 platform (Illumina, San Diego, United States).

### 2.9. Bioinformatics and data analysis.

After removing the adapters and primer sequences, the original sequences were assembled for each sample based on unique barcodes using QIIME (quantitative insights into microbial ecology). Split sequences from each sample were combined using Flash v.1.2.11. The valid labels of all samples were clustered using the Uparse software. Valuable sequences were classified into operational taxonomic units (OTUs) using USEARCH software, with 97% identification threshold as the cut-off point. For species annotation, representative bacterial sequences were matched against the SILVA database (version 138) and representative fungal sequences were classified using the UNITE database (version 8.0). Alpha diversity analysis was performed using Mothur software. Principal coordinate analysis (PCoA) and unweighted pairwise grouping algorithm clustering were performed using the R language. Tax4Fun was used to analyze the function of bacterial community. Fungal ecosystem analyses were performed using the FUNGuild database. Raw illumina sequencing data from the current study were submitted to the NCBI Sequence Read Archive (SRA) under the BioProject accession number PRJNA888947^2^ and PRJNA996339.^3^

### 2.10. Statistical analysis.

All experimental data were analyzed using Microsoft Excel 2019 and SAS 8.0. Statistical significance was set at *p* < 0.05.

## 3. Results

### 3.1. Isolation and identification of HSE-12 strains

**Figure 1:**
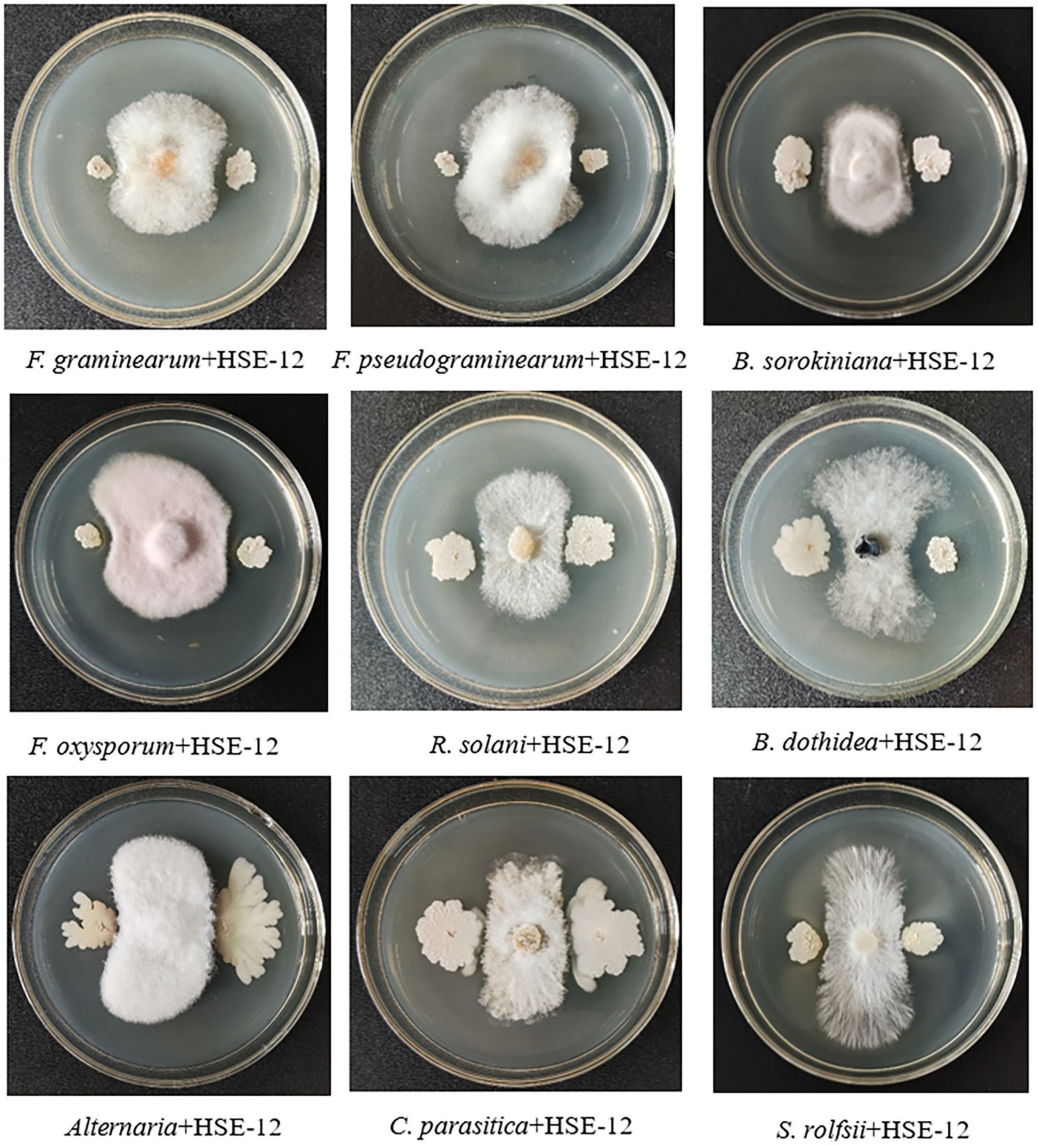
*In vitro* antagonism of strain HSE-12 against plant pathogens.

**Figure 2:**
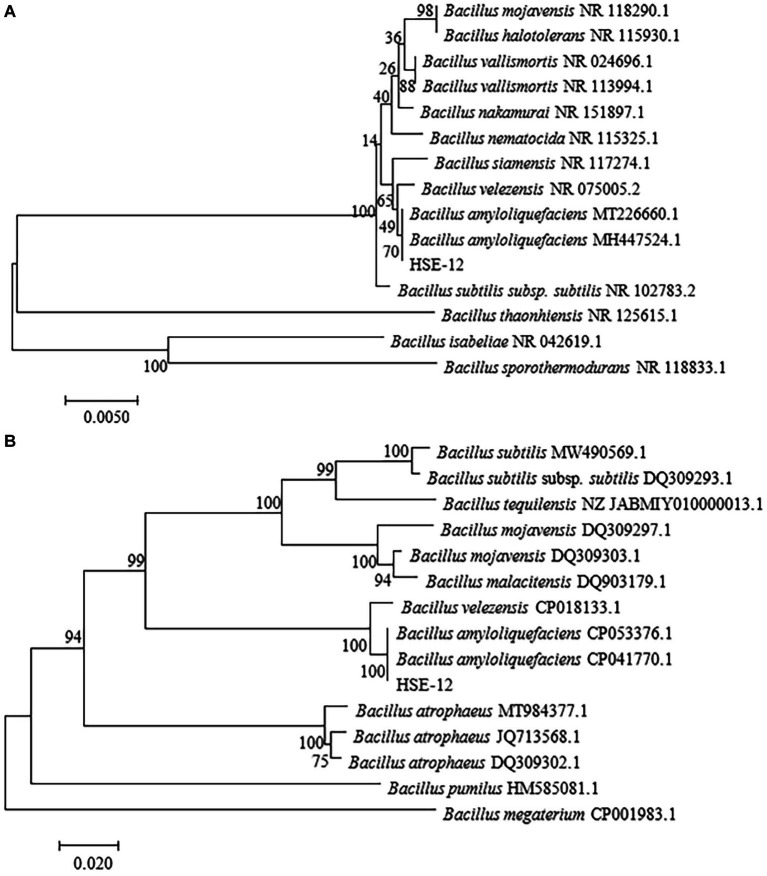
Neighbor-joining evolutionary tree constructed from 16S rDNA **(A)** and gyrB **(B)** sequence for strain HSE-12. Bootstrap values are shown on the nodes.

*In vitro* antagonism tests were carried out on bacteria isolated from peanut rhizosphere soil. Strain HSE-12 was able to inhibit the growth of *Sclerotium rolfsii, Fusarium oxysporum*, *Rhizoctonia solani*, *Fusarium graminearum*, *Fusarium pseudograminearum*, *Alternaria alternata*, *Botryosphaeria dothidea*, *Bipolaris sorokiniana* and *Cryphonectria parasitica*, as shown in Figure 1. The colony of HSE-12 is round, yellowish and opaque, with dry surface, wrinkles and irregular edges. The bacteria were rod-shaped, sporulated and Gram staining was positive. The glucose oxidation fermentation of HSE-12 strain is fermentation type, and it is positive in contact enzyme reaction, nitrate reduction, starch hydrolysis, gelatin liquefaction, citrate utilization, hydrogen sulfide production and V-P test, but negative in oxidase reaction, indole test and methyl red test.The 16S rDNA (A) and gyrB (B) sequence sequence of strain HSE-12 was analysed to construct a phylogenetic tree (Figure 2). The homology of 16S rDNA sequence between HSE-12 strain and known strain *Bacillus amyloliquefaciens* (MH 447524.1) was 99%, and the homology of gyrB sequence with *Bacillus amyloliquefaciens* (CP 053376.1) was 99%. HSE-12 strain was identified as *Bacillus amyloliquefaciens*.

### 3.2. Related indexes of antibacterial function of HSE-12 strain

#### 3.2.1. Determination of antagonistic fungal-associated enzyme activity

The activity of antagonist-related enzymes was determined for the production of strain HSE-12. After fermentation, chitinase, protease, cellulase and xylanase levels reached 17.64 ± 0.79 U/mL, 15.88 ± 0.27 U/mL, 35.95 ± 0.27 U/mL, and 44.69 ± 1.68 U/mL, respectively.

#### 3.2.2. GC–MS analysis and antifungal activity of VOCs

**Figure 3:**
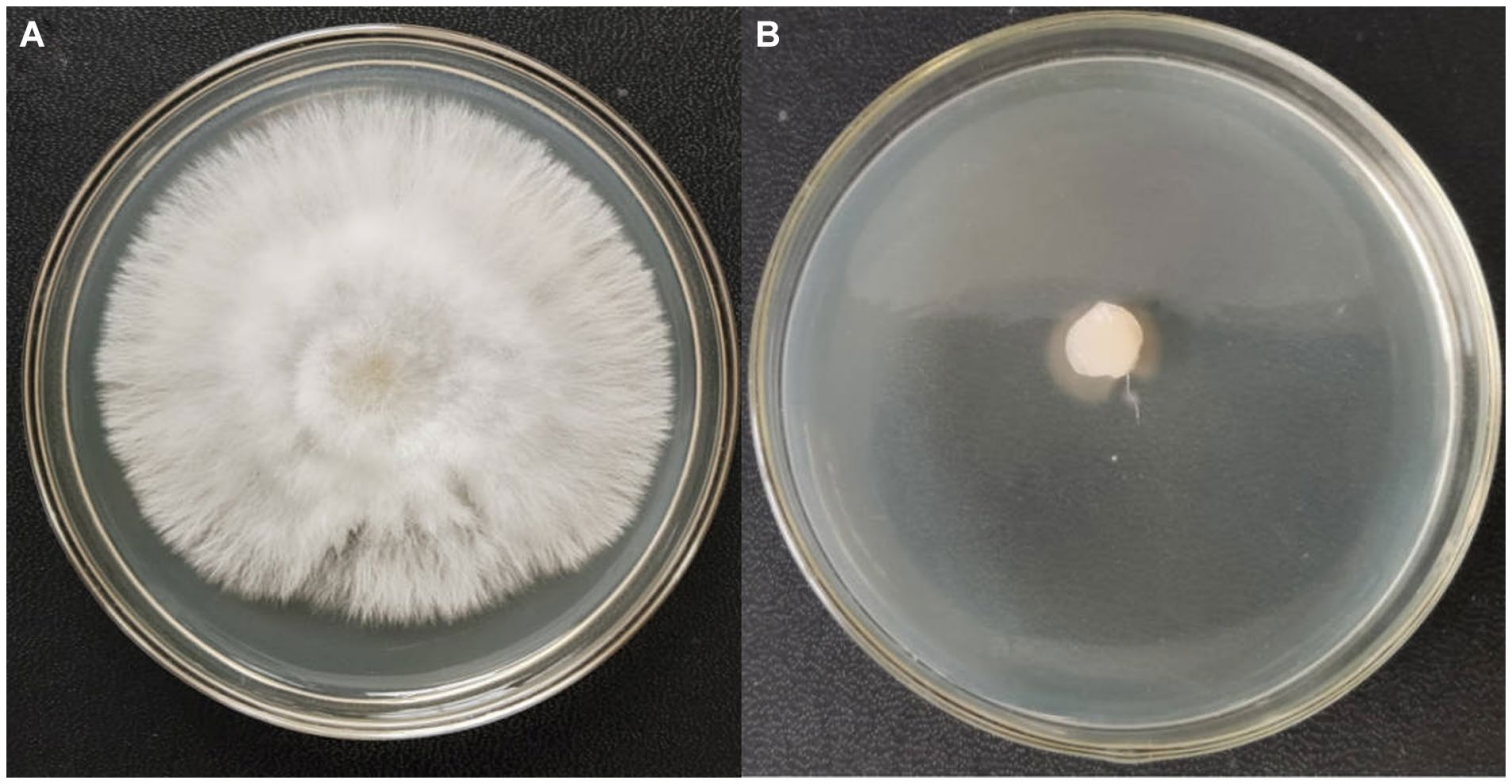
Inhibitory effect of VOCs produced by HSE-12 on *Sclerotium rolfsii*: **(A)** control treatment; **(B)** HSE-12 treatment.

The results of double plate antagonism assay were shown in Figure 3. The volatile fraction of the HSE-12 strain isolate was extracted using SPME and subsequently subjected to GC–MS to identify the volatile metabolites produced. Based on the chromatograms generated, a similar metabolic profile was observed in the isolates tested. Analysis of the mass spectra obtained from each peak observed the presence of 35 volatile compounds. Table 1 lists the compounds with biocontrol and probiotic effects, of which acetoin, 2,3-butanediol and N-methyl-N-nitroso-Ethanamine, were the first three major VOCs. 2,3-butanediol and acetoin were known to promote plant growth, antagonise fungi, induce resistance and improve plant tolerance (Wu et al., 2019; Silva Dias et al., 2021); ethanol inhibits *Staphylococcus aureus* and *Streptococcus pyogenes* (Rajendran et al., 2013); 2-nonanone, 3-methyl-2-pentanone, 5-methyl-2-hexanone and linalool have been shown to inhibit the growth of pathogenic bacteria (Li et al., 2015; Hou et al., 2021; Teliban et al., 2021).

**Table 1:** Types and relative contents of VOCs produced by HSE-12 strain.

Serial number	Rt (min)	Area (%)	Components	PGPR trait	Reference
1	1.158	0.05	Ethanol	Antagonism	Rajendran et al. (2013)
5	3.3	63.66	Acetoin	Antagonism and Growth-promoting	Wu et al. (2019)
6	3.683	0.02	3-methyl-2-Pentanone	Antagonism	Li et al. (2015)
8	4.253	0.17	2-methyl-Propanoic acid	Antagonism	Widada et al. (2021)
9	4.59	8.2	2,3-Butanediol	Antagonism	Silva Dias et al. (2021)
13	5.808	0.64	N-methyl-N-nitroso-Ethanamine	Antagonism	Hernández-León et al. (2015)
14	6.058	0.17	5-methyl-2-Hexanone	Antagonism	Li et al. (2015)
18	8.564	0.18	5-methyl-2-Heptanone	Antagonism	Morita et al. (2019)
20	9.14	0.13	2-pentyl-Furan	Antagonism	Li et al. (2018)
22	9.915	0.15	Linalool	Antagonism	Teliban et al. (2021)
24	10.544	0.08	2-Nonanone	Antagonism	Hou et al. (2021)

### 3.3. Growth promoting ability of HSE-12

In addition to its ability to antagonize fungi, HSE-12 also has the potential to promote plant growth. HSE-12 was found to have potassium solubilizing (10.54 ± 0.19 mg/L), phosphorus solubilizing (168.34 ± 8.06 mg/L) and nitrogen fixation (17.35 ± 2.34 mg/g) ability, and was able to secrete siderophore [(Ar-A)/Ar × 100%: 56%] which might induce plant growth.

### 3.4. Root colonization assay

The colonization of HSE-12 in peanut rhizosphere was determined by double antibiotic labeling method and plate counting method. As shown in Table 2, the survival number of HSE-12 in peanut rhizosphere was 10^5^ after adding bacterial liquid for 10 days, and it tended to be 10^4^ after 20 days, HSE-12 could colonize peanut rhizosphere.

**Table 2:** Population density of HSE-12 colonization in peanut rhizosphere.

Strain	Colony number cfu/g				
	10 d	20 d	30 d	40 d	50 d
HSE-12	3.00 × 10^5^	7.33 × 10^4^	1.23 × 10^4^	1.12 × 10^4^	1.24 × 10^4^

### 3.5. Seedling sampling and biomass and enzyme activity assay

Compare to CK, the dry weight, fresh weight and the length of the first lateral branch of peanut seedlings were significantly increased after treatment with HSE-12, which increased by 24.7, 41.9, and 36.4%, respectively, (Table 3; Supplementary Figure S1). The activities of SOD and POD in peanut leaves were significantly reduced, PAL activities were significantly increased, CAT and PPO activities were not significantly increased after treatment with HSE-12 strain (Table 4).

**Table 3:** Effect of strain HSE-12 on growth parameters of peanut seedlings.

	SH (cm)	LBH (cm)	FW (g)	DW (g)	NP (pcs)
CK	39.83 ± 2.43a	20.44 ± 0.79b	11.96 ± 1.3b	2.55 ± 0.21b	2.47 ± 0.28b
HSE-12	39.41 ± 1.22a	27.87 ± 1.86a	16.98 ± 1.23a	3.18 ± 0.34a	6.83 ± 2.98a

Values are means ± SD (*n* = 3). Means sharing a common letter within the same column are not signifcantly diferent at *p*<0.05. SH, the main stem height; LBH, the height of the first pair of lateral branches; FW, the fresh weight; DW, dry weight of the above-ground parts; NP, the number of pods.

**Table 4:** Effect of HSE-12 strain on defence enzyme activity of peanut seedling leaves.

	SOD (U·g^−1^FW)	POD (U·min^−1^·g^−1^FW)	CAT (U·min^−1^·g^−1^FW)	PPO (U·min^−1^·g^−1^FW)	PAL (U·min^−1^·g^−1^FW)
CK	218.62 ± 3.44a	84.47 ± 10.90a	44.86 ± 6.51a	3081.14 ± 147.47a	45.56 ± 10.08b
HSE-12	160.87 ± 8.19b	42.34 ± 2.53b	49.47 ± 4.62a	3281.66 ± 142.65a	74.08 ± 9.74a

Values are means ± SD (*n* = 3). Means sharing a common letter within the same column are not signifcantly diferent at *p*<0.05.

### 3.6. Soil chemical properties and enzyme activity

HSE-12 significantly increased the contents of alkali-hydrolyzable nitrogen, available phosphorus and the activities of sucrase, urease and catalase in peanut rhizosphere soil by 27.2, 36.5, 43.3, 50, and 69.8%, respectively (Table 5).

**Table 5:** Effect of HSE-12 strain on chemical properties and enzyme activity of peanut rhizosphere soil.

	AN (mg/kg)	AP (mg/kg)	AK (mg/kg)	SOM (g/kg)	Invertase (U)	Urease (U)	Phosphatase (U)	Catalase (U)
CK	57.17 ± 1.91b	44.48 ± 3.19b	86.54 ± 3.46a	40.05 ± 3.65a	14.61 ± 1.15b	0.20 ± 0.01b	3.97 ± 0.13a	0.63 ± 0.07b
HSE-12	72.72 ± 2.44a	60.7 ± 2.35a	82.84 ± 13.82a	41.91 ± 5.11a	20.94 ± 0.56a	0.30 ± 0.02a	4.12 ± 0.25a	1.07 ± 0.10a

Values are means ± SD (*n* = 3). Means sharing a common letter within the same column are not signifcantly diferent at *p*<0.05.

### 3.7. Analysis of the diversity and structure of bacterial and fungi communities

The diversity of the peanut rhizosphere soil bacterial and fungi community was shown in Table 6. The ACE, Chao1 and Shannon indices of the bacterial community in the HSE-12 treatment were significantly higher than the control. ACE, Chao1 and Shannon index of fungal community in HSE-12 treatment were slightly higher than those in control (but not significant). This indicates that the HSE-12 treatment significantly increased the diversity and richness of the soil bacterial community between the roots of peanut.

**Table 6:** Diversity and richness indices of the bacterial communities of the HSE-12 treatment and control.

Microbial community	Peanut fields	ACE	Chao 1	Shannon	Coverage
Bacteria	CK	2758.2 ± 207.90b	2722.51 ± 200.10b	6.13 ± 0.03b	0.98a
	HSE-12	3115.25 ± 109.94a	3107.59 ± 122.68a	6.4 ± 0.04a	0.98a
Fungi	CK	730.52 ± 108.18a	730.62 ± 95.04a	3.33 ± 0.68a	0.98a
	HSE-12	753.22 ± 23.97a	746.96 ± 23.24a	3.79 ± 0.49a	0.98a

Values are means ± SD (*n* = 3). Means sharing a common letter within the same column are not signifcantly different at *p*<0.05.

**Figure 4:**
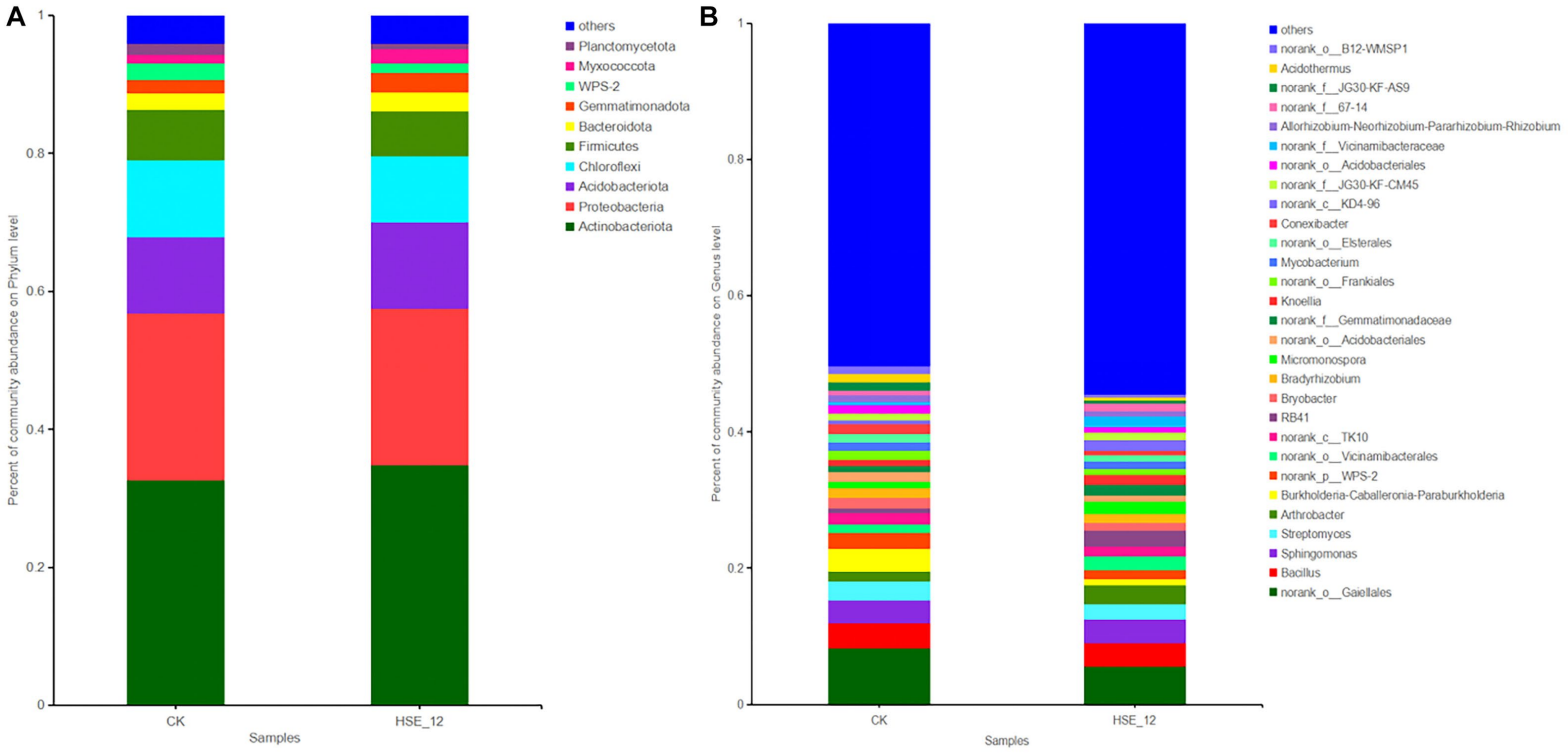
Relative abundance of bacterial communities at the phylum level **(A)** and genus level **(B)** in peanut rhizosphere soils from HSE-12 and control treatments.

For bacteria, at the phylum level, the dominant bacterial composition of the CK and HSE-12 groups was essentially the similar, being Actinobacteriota (32.6, 34.8%), Proteobacteria (24.2, 22.7%), Acidobacteriota (11.1, 12.6%), and Chloroflexi (11.2, 9.6%), with the relative abundance of Actinobacteriota being the greatest in each treatment group (Figure 4A).

At the genus level, the relative abundance of 29 genera (at least in one treatment group) was greater than 1%. The top three genera of CK were *norrank_o_gaiellales* (8.3%), *Bacillus* (3.7%), and *Burkholderia-Caballeria-Paraburkholderia* (3.4%). The top three genera in HSE-12 group were *norrank_o_gaiellales* (5.5%), *Sphingomonas* (3.5%), and *Bacillus* (3.5%) (Figure 4B).

**Figure 5:**
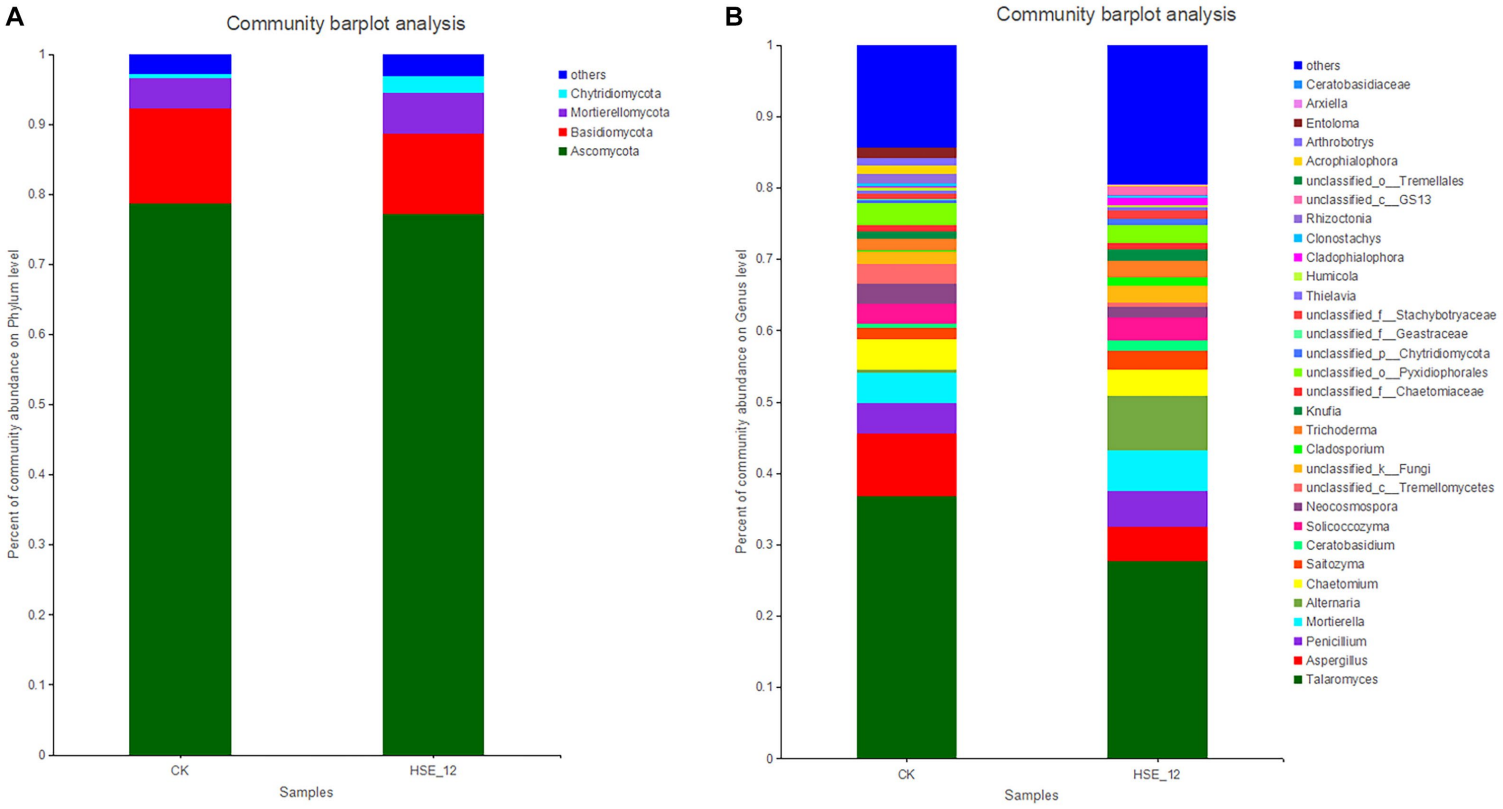
Relative abundance of fungi communities at the phylum level **(A)** and genus level **(B)** in peanut rhizosphere soils from HSE-12 and control treatments.

The fungi in CK and HSE-12 treatment were mainly composed of four phyla, namely Ascomycota (78.7, 77.3%), Basidiomycota (13.5, 11.5%), Mortierellomycota (4.3, 5.8%), and Chytridiomycota (0.7, 2.4%) (Figure 5A). At the genus level, the relative abundance of 32 genera (at least in one treatment group) was greater than 1%. The top three genera of CK were *Talaromyces* (36.8%), *Aspergillus* (8.8%), and *Penicillium* (4.3%). The top three genera in HSE-12 group were *Talaromyces* (27.7%), *Alternaria* (7.5%), and *Mortierella* (5.8%) (Figure 5B). HSE-12 changed the composition of peanut rhizosphere bacterial and fungi community.

### 3.8. PCoA and hierarchical clustering analysis of soil bacterial community

**Figure 6:**
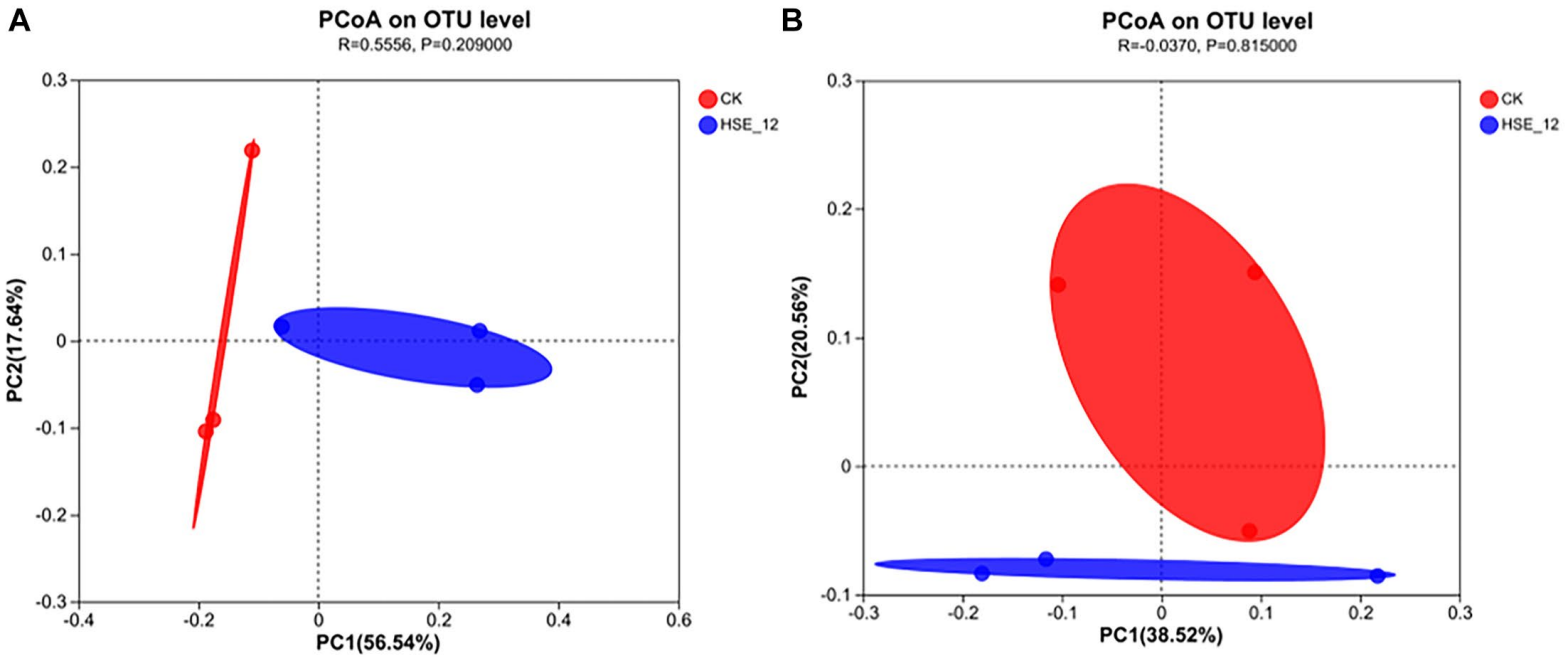
PcoA analysis of bacterial **(A)** and fungi **(B)** communities in peanut rhizosphere soil for HSE-12 treatment and control treatment.

Principal coordinate analysis (PCoA) was used to analyse the differences in microbial diversity between treatments (Figure 6). The results showed that for bacterial and fungal communities, the HSE-12 strain treatment samples were completely separated from CK, indicating a significant difference in bacterial community diversity between the two treatments. For bacteria, the PC1 value was 56.54% and the PC2 value was 17.64%, which means that these two axes explained 74.18% of the difference in diversity. For fungi, the PC1 value was 38.52% and the PC2 value was 20.56%, which means that these two axes explained 59.08% of the difference in diversity.

### 3.9. Prediction and analysis of bacterial and fungi community function

**Figure 7:**
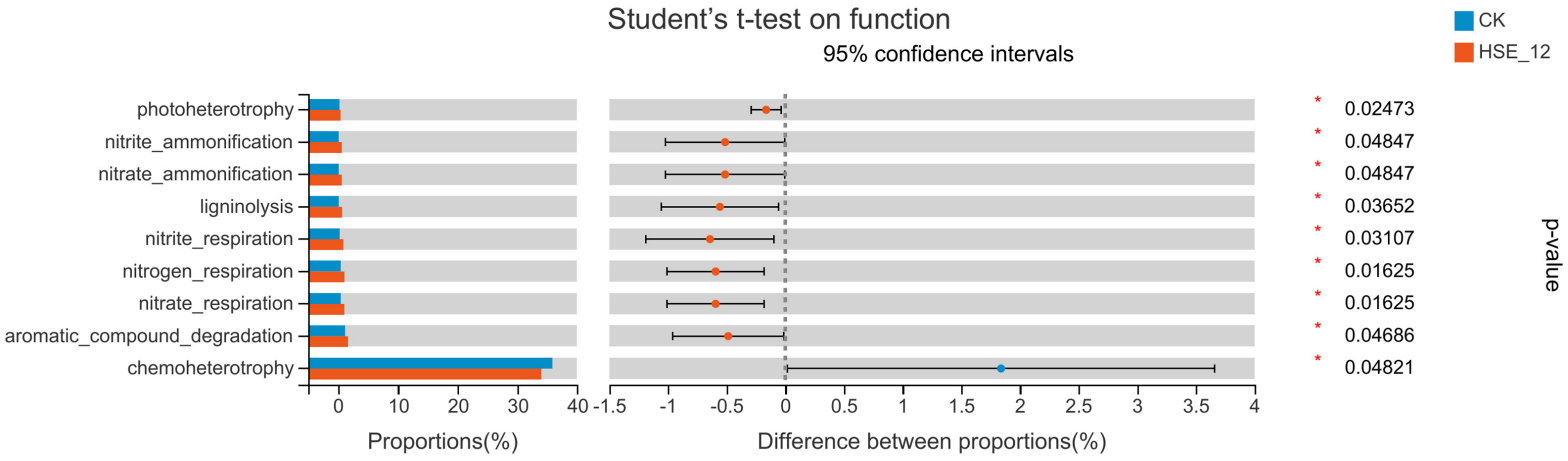
Functional prediction of bacterial communities in peanut rhizosphere soil from HSE-12 and control treatments.

The function of bacterial community was predicted by FAPROTAX software (Figure 7). The results showed that the bacterial communities related to carbon and nitrogen cycle function in HSE-12 strain treatment group, such as nitrate_ammonification, nitrate_ammonification and ligninolysis, were significantly higher than those in CK treatment group. Therefore, HSE-12 may promote plant growth by enriching bacterial communities related to carbon and nitrogen cycle function.

**Figure 8:**
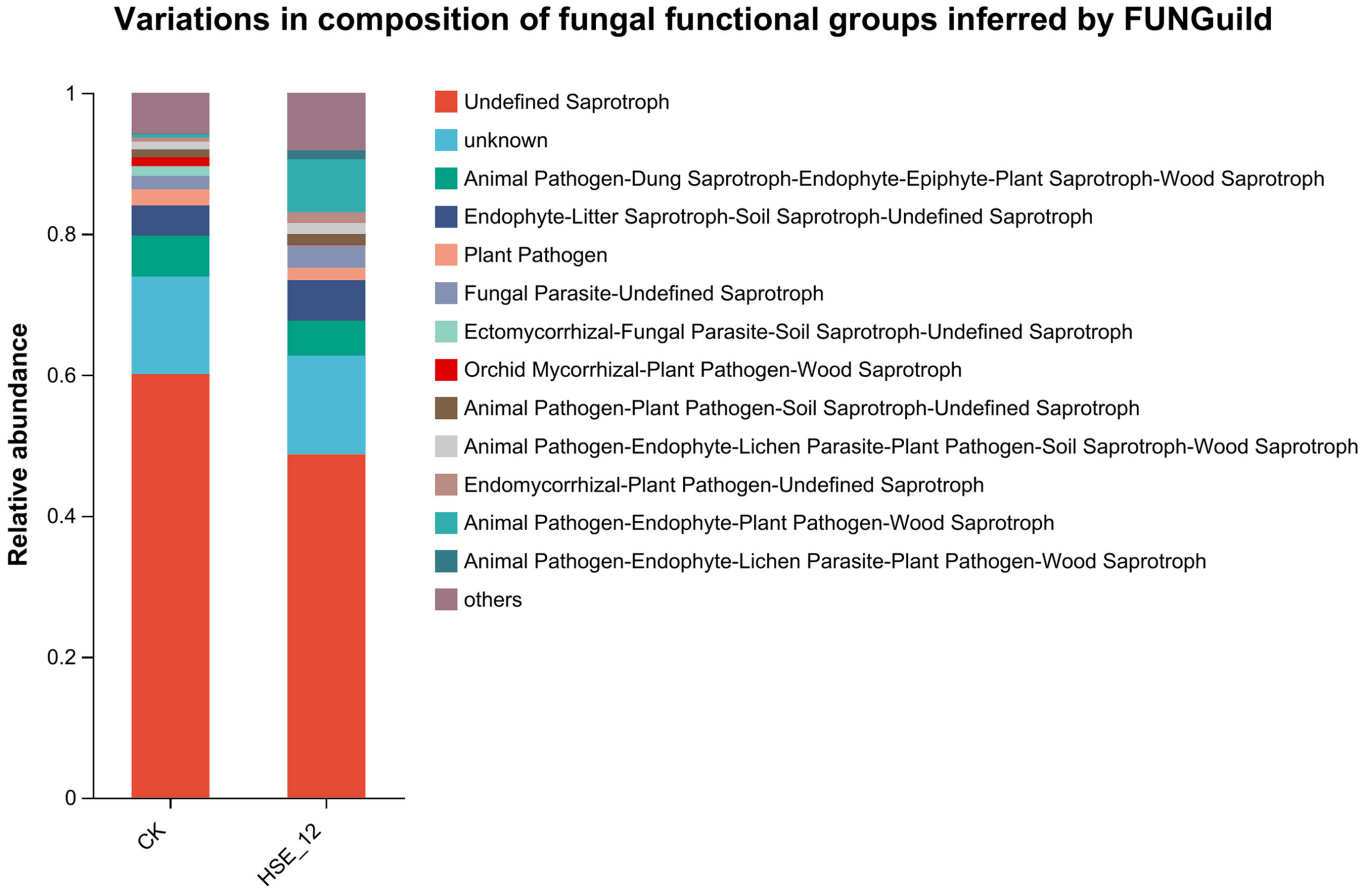
Functional prediction of fungi communities in peanut rhizosphere soil from HSE-12 and control treatments.

Function of fungal community was annotated by FUNGuild (Figure 8). In CK treatment, the top three functions of relative abundance of fungal communities were Undefined Saprotroph (60.1%), Animal Pathogen-Dung Saprotroph-Endophyte-Epiphyte-Plant Saprotroph-Wood Saprotroph (5.8%) and Endophyte-Litter Saprotroph-Soil Saprotroph-Undefined Saprotroph (4.3%). In HSE-12 strain treatment, the top three functions of relative abundance of fungal communities were Undefined Saprotroph (48.7%), Animal Pathogen-Endophyte-Plant Pathogen-Wood Saprotroph (7.5%), and Endophyte-Litter Saprotroph-Soil Saprotroph-Undefined Saprotroph (5.8%). In addition, it was found that the relative abundance of plant pathogens in the fungal community treated by HSE-12 was lower than that of CK treatment group.

## 4. Discussion

Plant growth promoting rhizobacteria can not only promote plant growth, increase crop yield and control plant diseases and insect pests, but also has specific microecological functions. It is an important biological control method with great potential (Song et al., 2021). It has been shown that many microorganisms including *Azospirillum, Azotobacter, Alcaligenes, Arthrobacter, Burkholderia, Bacillus, Enterobacter, Klebsiella, Pseudomonas*, and *Serratia* have a plant growth promoting effect (Mohanty et al., 2021). *Bacillus* is one of the application prospects in the agricultural field as a biological control agent for biological diseases. It is estimated that *Bacillus* alone accounts for half of the commercial bacterial biological control agents. As one of the most studied microorganisms, some strains of *Bacillus* have shown their potential as plant growth-promoting bacteria and have been proved to have antagonistic activities against several plant pathogenic microorganisms (Dame et al., 2021). For instance, Nabi et al. found that *Bacillus aryabhattai* strain SRB02 was able to control tomato wilt caused by *Fusarium* sp. *lycopersici* and had the ability to promote plant growth by regulating endogenous hormone and amino acid levels (Syed Nabi et al., 2021). *Bacillus* sp. WM13-24 promoted increased photosynthetic capacity, dry weight and fresh weight of ryegrass by regulating phytohormone distribution, and enhanced drought tolerance of ryegrass by increasing antioxidant enzyme activity and modulating ABA signal transduction (He et al., 2021). In recent research, it was found that the application of *Paraburkholderia fungorum* strain BRRh-4 and *Delftia* sp. strain BTL-M2 significantly improved the germination, growth and yield of rice seeds, and could reduce the demand for nitrogen, phosphorus and potassium fertilizer by 50% without affecting the yield. This may be related to the diversity, structure and characteristics of rhizosphere and rhizosphere soil flora regulated by two plant probiotics. This study confirmed for the first time that the application of plant growth-promoting bacteria can significantly promote the growth, yield and bacterial community diversity of rice (Islam et al., 2023). In this study, HSE-12 significantly increased the dry weight, fresh weight and the height of the first pair of lateral branches of peanut. The main reason may be that HSE-12 significantly increased the contents of alkali-hydrolyzable nitrogen, available phosphorus and the activities of sucrase, urease and catalase in peanut rhizosphere soil. In addition, HSE-12 was found to have the ability to produce hormone. The yields of IAA, GA3 and ZA were determined by high performance liquid chromatography, which were 0.97 μg/mL, 0.64 μg/mL, and 0.59 μg/mL, respectively. Refer to supplementary S1 materials for determination methods. It is possible that HSE-12 promoted the growth of peanut through hormone production.

Biological stress in plants is brought about by living organisms such as bacteria, fungi and insects, for example. This stress directly affects the uptake of nutrients by the host and in severe cases can lead to plant death. PGPR is known to mitigate the damage caused by biotic stress to plants through a variety of mechanisms, including bacteriocins, antibiotics, volatile organic compound (VOC) production and cleavage through extracellular enzymes. Ghadamgahi et al. found that *Pseudomonas aeruginosa* FG106 was antagonistic to a wide range of pathogenic bacteria, can produce proteases and lipases, and also had the ability to solubilizing phosphorus and produce iron carriers, ammonia, IAA and hydrogen cyanide (HCN), thereby promoting plant growth and facilitating biological control (Ghadamgahi et al., 2022). Rangel-Montoya et al. screened two strains of *Bacillus amylolyticus*, BsA3MX and BsC11MX, which inhibited *M. phaseolina in vitro* by up to 66.8% through the combined action of volatile and diffusible compounds. In addition, they were able to produce iron carriers and IAA and had ACC deaminase activity and phospholysis. Furthermore, in greenhouse trials on cowpea plants (*Vigna unguiculata* L.), strain BsA3MX reduced damage caused by *M. phaseolina* and had a significant pro-growth effect (Rangel-Montoya et al., 2022). In this study, HSE-12 inhibited the growth of *Sclerotium rolfsii in vitro* through the combined action of volatile and non-volatile compounds, and it had broad-spectrum resistance. HSE-12 can produce volatile compounds such as acetoin and 2,3-butanediol to inhibit the growth of pathogenic bacteria and promote plant growth.

The main function of soil enzymes is to decompose and convert nutrient forms that cannot be directly utilized by plants into forms that are easily absorbed and utilized by plants. They are also involved in many biochemical processes, including the synthesis and decomposition of humus, the hydrolysis of animal and plant residues, and various redox reactions of organic and inorganic compounds in soil (De Corato, 2021). Here, we found that the application of HSE-12 significantly increased the activities of urease, sucrase and catalase, and decreased the activities of POD and SOD in leaves. The above results indicated that HSE-12 could enhance the conversion and utilization of soil nutrients, reduce the stress pressure of plants and the level of peroxides in plants, resulting in the decline of antioxidant enzyme activities, promotion of plant growth.

Illumina MiSeq sequencing analysis showed that the inoculated strain HSE-12 significantly increased the diversity and richness of soil bacterial community. The HSE-12 increased the relative abundance of beneficial bacteria such as *Sphingomonas*, *Arthrobacter*, *RB41*, and *Micromonospora*. *Sphingomonas* is a genus of bacteria that is widely distributed in nature, commonly found in soil and aquatic environments, and has been shown to inhibit infection with *Pseudomonas syringae*, *Fusarium oxysporum* and *Fusarium insipidum* and to promote plant growth (Wachowska et al., 2013; Luo et al., 2020). *Arthrobacter* is a group of Actinomyces known to inhibit plant pathogens. For example, four arthrobacter strains isolated from the compost inhibit the growth of *Fusarium sambucinum*, *Botrytis cinerea*, *Pythium sulcatum*, *Verticillium dahliae*, and *Alternaria alternata*. The secondary metabolites of Arthrobacter play a key role in their antagonism (Ramlawi et al., 2021). *RB41* is also a bacterial genus of Acinetobacter that may help maintain metabolism of soil organisms under long-term low nutritional conditions (Jiang et al., 2021). *Micromonospora* is a kind of rare actinomycetes, widely distributed in soil, sea, animals and plants, and has long been considered as an important source of antibiotics. It is found that several micromonospora can inhibit several pathogens *in vitro* and in plants, and they can also produce antibacterial and antifungal compounds to protect plants from pathogens. These findings indicate that micromonospora has the potential to become a biocontrol agent (Cui et al., 2020).

As for fungi, the HSE-12 decreased the relative abundance of *Talaromyces* in peanut rhizosphere soil compared with CK. The species of this genus is an important pollutant in food industry, a producer of mycotoxin and a pathogen of human beings in medicine (Stošić et al., 2020; Liu et al., 2021; Xu et al., 2021b). HSE-12 strain reduced the relative abundance of pathogenic fungi such as *Aspergillus*, *Neocosmospora* and *Rhizoctonia*. The highly diverse *Aspergillus* fungus is a well-known agricultural fungal disease and a producer of various fungal toxins that threaten food safety worldwide. *Aspergillus niger* causes crown rot in peanuts and is contagious throughout the peanut growing season, especially during the sowing and early growing season. It mainly infects the base of the stem and causes direct seed death, resulting in high yield losses in peanuts (Xu et al., 2021a). *Neocosmospora* is a fungus that is widespread in soil, plants, air and water. Given its importance as a plant pathogen, it has been used as a model organism in plant molecular pathology, and the fungus causes basal rot of peanut (Sandoval-Denis et al., 2019). HSE-12 increased the relative abundance of beneficial fungi such as *Penicillium*, *Cladosporium* and *Trichoderma*. *Trichoderma* fungi can control the growth and proliferation of harmful pathogens through a variety of mechanisms including fungal parasitism, antibiotic production and competition for nutrients or space (Mukhopadhyay and Kumar, 2020). Therefore, HSE-12 increased the relative abundance of beneficial microorganisms and decreased the relative abundance of pathogenic microorganisms in peanut rhizosphere.

## Data availability statement

The datasets presented in this study can be found in online repositories. The names of the repository/repositories and accession number(s) can be found at: https://www.ncbi.nlm.nih.gov/, PRJNA888947 https://www.ncbi.nlm.nih.gov/, PRJNA996339.

## Author contributions

HL: Data curation, Writing – original draft, Writing – review & editing, Project administration. CL: Data curation, Project administration, Writing – original draft, Writing – review & editing. XS: Data curation, Writing – review & editing. JL: Writing – review & editing. PZ: Writing – review & editing. FS: Writing – review & editing. ZG: Writing – review & editing. XL: Conceptualization, Funding acquisition, Resources, Writing – original draft, Writing – review & editing.

## Footnotes

^1^https://www.ncbi.nlm.nih.gov

^2^https://www.ncbi.nlm.nih.gov/bioproject/PRJNA888947

^3^https://www.ncbi.nlm.nih.gov/bioproject/PRJNA996339
