# Supplementary material for: Isolation and identification of antagonistic Bacillus amyloliquefaciens HSE-12 and its effects on peanut growth and rhizosphere microbial community
Source: Front Microbiol. 2023 Oct 12;14:1274346. doi: 10.3389/fmicb.2023.1274346 (PMC10601714; doi:10.3389/fmicb.2023.1274346)
Supplement: Supplementary file 1 [file Data_Sheet_1.docx]

Materials and methods

Bacterial cultures (NFb) in the exponential growth phase were separated into several 20 mL. The conditions of IAA HPLC were as follows: mobile phase A was methanol, mobile phase B was 1% acetic acid, 40% A+60% B; The conditions of GA3 HPLC were as follows: mobile phase A was methanol, mobile phase B was 0.1% phosphoric acid aqueous solution, 35% A+65% B; The conditions of ZA HPLC were as follows: mobile phase A was methanol, mobile phase B was water, and 50% A+50% B. IAA, GA3 and ZA were determined at UV wavelengths of 275, 210 and 254 nm, respectively.
