# Supplementary figures and images for: Isolation and identification of antagonistic Bacillus amyloliquefaciens HSE-12 and its effects on peanut growth and rhizosphere microbial community
Source: Front Microbiol. 2023 Oct 12;14:1274346. doi: 10.3389/fmicb.2023.1274346 (PMC10601714; doi:10.3389/fmicb.2023.1274346)

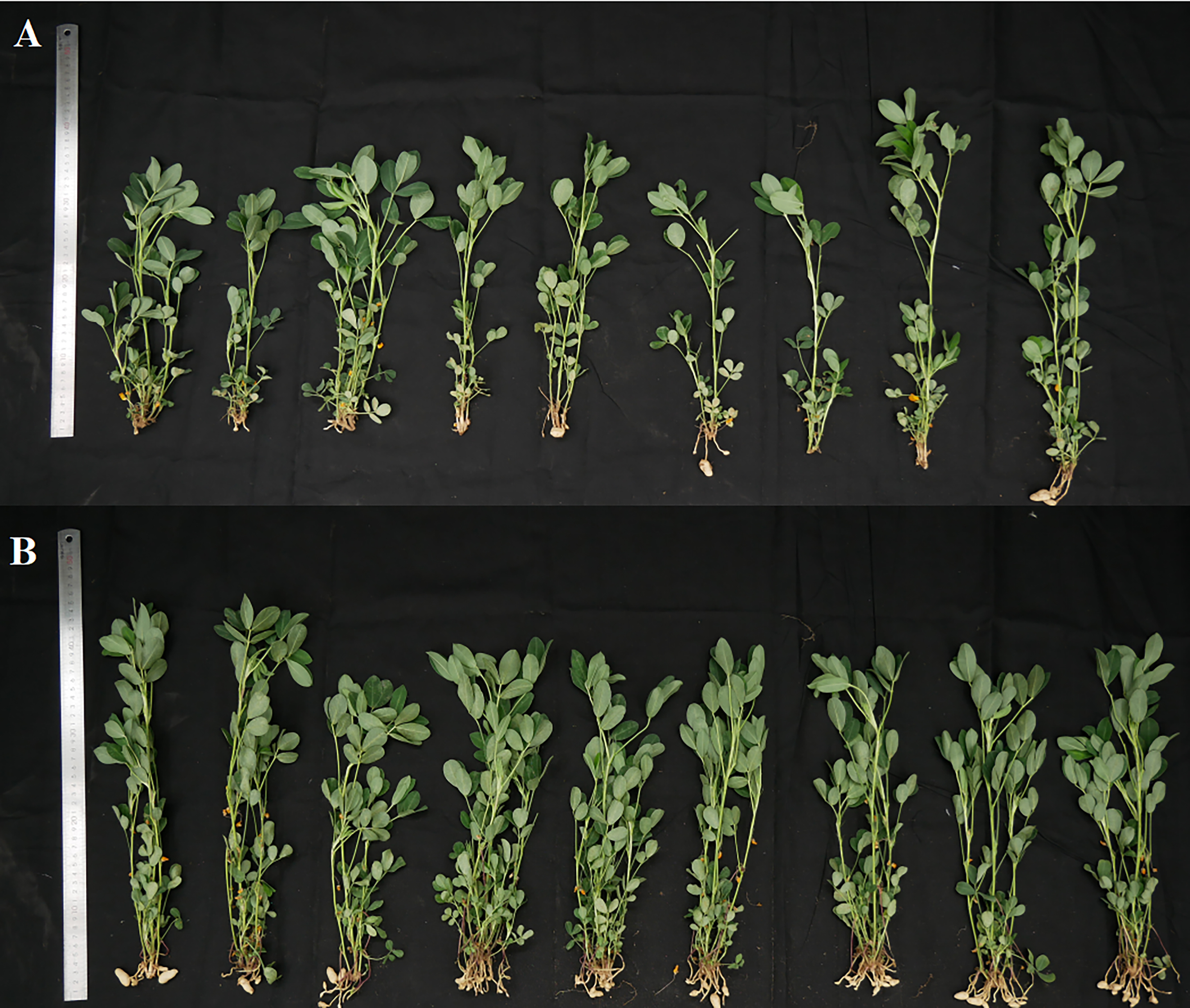

Supplement: Supplementary file 2 [file Image_1.JPEG]
